# The Enhanced Metastatic Potential of Hepatocellular Carcinoma (HCC) Cells with Sorafenib Resistance

**DOI:** 10.1371/journal.pone.0078675

**Published:** 2013-11-11

**Authors:** Ariel Ka-Man Chow, Lui Ng, Colin Siu-Chi Lam, Sunny Kit-Man Wong, Timothy Ming-Hun Wan, Nathan Shiu-Man Cheng, Thomas Chung-Cheung Yau, Ronnie Tung-Ping Poon, Roberta Wen-Chi Pang

**Affiliations:** 1 Centre for Cancer Research, LKS Faculty of Medicine, The University of Hong Kong, Hong Kong SAR, China; 2 Department of Surgery, LKS Faculty of Medicine, The University of Hong Kong, Hong Kong SAR, China; Osaka University Graduate School of Medicine, Japan

## Abstract

Acquired resistance towards sorafenib treatment was found in HCC patients, which results in poor prognosis. To investigate the enhanced metastatic potential of sorafenib resistance cells, sorafenib-resistant (SorR) cell lines were established by long-term exposure of the HCC cells to the maximum tolerated dose of sorafenib. Cell proliferation assay and qPCR of ABC transporter genes (ABCC1-3) were first performed to confirm the resistance of cells. Migration and invasion assays, and immunoblotting analysis on the expression of epithelial to mesenchymal transition (EMT) regulatory proteins were performed to study the metastatic potential of SorR cells. The expression of CD44 and CD133 were studied by flow cytometry and the gene expressions of pluripotency factors were studied by qPCR to demonstrate the enrichment of cancer stem cells (CSCs) in SorR cells. Control (CTL) and SorR cells were also injected orthotopically to the livers of NOD-SCID mice to investigate the development of lung metastasis. Increased expressions of ABCC1-3 were found in SorR cells. Enhanced migratory and invasive abilities of SorR cells were observed. The changes in expression of EMT regulatory proteins demonstrated an activation of the EMT process in SorR cells. Enriched proportion of CD44^+^ and CD44^+^CD133^+^ cells were also observed in SorR cells. All (8/8) mice injected with SorR cells demonstrated lung metastasis whereas only 1/8 mouse injected with CTL cells showed lung metastasis. HCC cells with sorafenib resistance demonstrated a higher metastatic potential, which may be due to the activated EMT process. Enriched CSCs were also demonstrated in the sorafenib resistant cells. This study suggests that advanced HCC patients with acquired sorafenib resistance may have enhanced tumor growth or distant metastasis, which raises the concern of long-term sorafenib treatment in advanced HCC patients who have developed resistance of sorafenib.

## Introduction

Hepatocellular carcinoma (HCC) is the fifth leading cancer in men and the seventh leading cancer in women with a total of 0.7 million new cases worldwide [1]. Only a minority of HCC patients are eligible to locoregional treatments including surgical resection [2], [3]. In addition, tumor response rate of HCC patients towards systemic chemotherapy is low and chemoresistance can easily develop [4]–[7]. HCC is still the second and the sixth leading cause of cancer-related deaths in men and women, respectively, with over half a million deaths worldwide [1] and the overall 5-year survival rate of patients with advanced HCC is below 10% [8]. Therefore, it is of utmost importance to develop new medical treatment especially for advanced HCC patients.

Sorafenib is an oral multikinase inhibitor, approved for the treatment of advanced renal cell carcinoma and HCC by the U.S. Food and Drug Administration and the European Medicine Agency, targeting on Raf, epidermal growth factor receptor (EGFR), vascular endothelial growth factor receptor (VEGFR), platelet-derived growth factor receptor (PDGFR), FMS-like tyrosine kinase-3 (Flt-3) and c-kit [9]. Sorafenib treatment was found to be effective in inhibiting tumor growth and angiogenesis in HCC by two large-scale, randomized, placebo-controlled studies and the median overall survival rate is approximately 3 months longer in the sorafenib treatment group [10], [11]. Recent reports on patients with long-term treatment of sorafenib demonstrated that only manageable adverse effects with mild-to-moderate in severity were reported in patients with advanced non-small-cell lung cancer [12], advanced renal cell carcinoma [13], and advanced HCC [14].

Although sorafenib is a potent anti-cancer drug in treating patients with advanced HCC, many patients still develop acquired resistance to sorafenib [15]. A number of recent studies also reported that many different pathways are involved in the development of sorafenib resistance [16]. Chen et al. demonstrated that the activation of the PI3K/Akt signaling pathway mediates the acquired sorafenib resistance in Huh7 cells [17]. In addition, the expression level of EGFR was found to anticipate the efficacy of sorafenib treatment [18] and blocking of EGFR and HER-3 phosphorylation sensitizes HCC cell response to sorafenib [19]. Enrichment of cancer stem cells (CSCs) may also contribute to sorafenib resistance. Label-retaining liver cancer cells, which represent a novel subpopulation of CSCs, were found to be resistant to sorafenib and these cells might contribute to disease recurrence in HCC [20].

In view of the possibility of acquired sorafenib resistance with long-term sorafenib treatment, the adverse effects brought by the resistant cells were not known completely. In this study, three HCC cell lines with sorafenib resistance were induced by long-term cultured with sorafenib at the maximal tolerated dose. Changes in cell morphology and the migratory and invasive abilities of sorafenib resistant (SorR) cells were studied. We further provide evidence to support that these changes were caused by the activation of the EMT process. Enrichment of CD44^+^ and CD44^+^CD133^+^ subpopulations of CSCs and enhanced expression of pluripotency factors further suggested the possibility of tumor recurrence or metastasis caused by SorR cells. Finally, animal study was performed to demonstrate the increased incidence of lung metastasis after inoculating SorR cells orthotopically to the liver of mice. This study suggests that a higher metastatic potential of HCC cells might be developed in HCC patients with acquired sorafenib resistance, which brings out the concern of sorafenib treatment in advanced HCC patients.

## Materials and Methods

### Drugs and Reagents

Sorafenib tosylate was provided by Bayer HealthCare Pharmaceuticals Inc. All reagents were purchased from Sigma-Aldrich (St. Louis, MO, USA), unless specified below.

### Cell Culture and Treatment

Human hepatoma PLC/PRF/5 (CRL-8024) (ATCC, Manassas, VA), HepG2 (HB-8065) (ATCC) and MHCC97L (Liver Cancer Institute, Fudan University, Shanghai, China [21]) cells were maintained in DMEM (Life Technologies, Carlsbad, CA) containing 10% FBS (Life Technologies) and 1% Penicillin/Streptomycin (Life Technologies), at 37°C humidified incubator with 5% CO_2_ in the air. PLC/PRF/5 cells were stably transfected with luciferase expressing construct for the ease of detection in the *in vivo* study. For the development of sorafenib resistant cells (SorR), cells were treated with 1 µM sorafenib and the concentration of sorafenib was increased by 10% every two weeks until the maximum tolerated doses have been reached and sorafenib resistance have been developed. Equal volume of DMSO was added to the control cells (CTL).

### Cell Proliferation

The difference in sensitivity towards sorafenib treatment was first examined by MTT assay as previously described [22]. Briefly, CTL and SorR cells were plated in 96-well culture plates for 24 hours and media were replaced with culture medium with the indicated concentrations (0–14 µM) of sorafenib. After 72 hours, viability was assessed with the addition of MTT solution (1 mg/ml) (Life Technologies). The percentage of surviving cells was determined by dividing the average absorbance of sorafenib-treated cells by the average absorbance of untreated cells from 3 replicate samples.

### Actin Staining

CTL and SorR cells were plated in 8-well Millicell EZ slides (Millipore, Billerica, MA), at 1000 cells per well. 24 hours after attachment, cells were fixed with 4% paraformaldehyde at room temperature for 5 min. Cells were then permeabilized with 0.2% Triton X-100. Actin filaments were detected by incubating with 50 µg/ml TRITC conjugated phalloidin for 1 hour at room temperature. After washing with PBS for several times, actin filaments were visualized using a fluorescence microscope.

### Migration and Invasion Transwell Assay

CTL and SorR cells were plated in top chambers of 24-well transwell plates with 8 µm pores (Corning) and 24-well BioCoat™ Matrigel™ Invasion chamber (BD Biosciences) at 1×10^5^ cells per well in DMEM with 1% FBS, for the study of migration and invasion, respectively. 10% FBS was used as chemoattractant. After 48 hours incubation, migrated or invaded cells were stained with 0.2% crystal violet. The numbers of migrated and invaded cells in four fields were counted under 100× magnification and the average numbers of migrated and invaded cells were counted.

### Immunoblotting

Total proteins were obtained by lysing CTL and SorR cells with ice-cold RIPA buffer containing 150 mM NaCl, 1 mM EDTA, 1% NP-40, 0.25% sodium deoxycholate, 1 mM PMSF, and protease inhibitor cocktail (Roche Diagnostics, Penzbery, Germany) in 50 mM Tris-HCl buffer, pH 7.4. As for the nuclear fraction, nuclear protein was obtained using the nuclear/cytosol fractionation kit (Biovision, Milpitas, CA) according to the manufacturer’s instruction. Total and nuclear protein were subjected to immunoblotting as previously described [22]. Equal amount of protein was loaded onto a 10% SDS-polyacrylamide gel under reducing condition and transferred to PVDF membrane (Amersham Bioscience, Piscataway, NJ). For the total protein fraction, blots were probed with the following antibodies: E-cadherin, Snail, N-cadherin (Novus Biologicals, Littleton, CO) and Vimentin (Abcam) and expression of β-actin (Sigma-Aldrich) was used as loading control. As for the nuclear fraction, blots were probed with the following antibodies: β-catenin (BD Biosciences), Smad2 (Cell signaling) and Smad3 (Cell signaling), and the expression of nuclear matrix protein p84 (GeneTex, San Antonio, TX) was used as loading control. After probing with horseradish peroxidase-conjugated secondary antibodies, membranes were developed with the Immobilon Western Chemiluminescent HRP substrate system (Millipore).

### Flow Cytometry on CD44 and CD133 Distribution

Harvested CTL and SorR cells were stained with CD44-FITC (BD Pharmingen) and CD133-APC (Miltenyl Biotec, Auburn, CA) antibodies in PBS with 1% BSA and 2 mM EDTA for 15 min at room temperature in dark. Presence of CD44+ and CD133+ cells were determined by Cytomics FC 500 flow cytometer (Beckman Coulter, CA) and analyzed using FlowJo (version 8.7, Tree Star, Inc.).

### Quantitative Real Time PCR (qPCR)

Total RNA from CTL and SorR cells was extracted using Trizol Reagent (Life Technologies) and purified using the PureLink RNA Mini Kit (Life Technologies) according to the manufacturer’s instructions. The concentration of RNA was quantified by a NanoDrop spectrophotometer (Thermo Scientific, Wilmington, DE). Complementary DNA (cDNA) was synthesized from 1 µg RNA using superscript III reverse transcriptase (Life Technologies). Primers (Life Technologies) used were listed in Table 1. qPCR reactions were carried out using SYBR Green PCR master mix (Life Technologies) according to the manufacturer’s instruction and run on a real-time PCR 7900 HT system (Applied Biosystems, Foster City, CA). The expression level of β-actin was used as internal control.

**Table 1:** Sequences of primer pairs.

Gene	Sequence
*ABC transporter proteins*	
ABCC1	F: 5′-CCA TCC ACG ACC CTA ATC CC-3′
	R: 5′-ACT TGT TCC GAC GTG TCC TC-3′
ABCC2	F: 5′-AGG TCA TCC TTT ACG GAG AAC A-3′
	R: 5′-GTC CAG GAA TGA GGA ATT CCA AAA A-3′
ABCC3	F: 5′-GAC TCA GGC CAG TGT GTC TC-3′
	R: 5′-GGT GCC ACT GTG TAT GGT GA-3′
*Pluripotent factors*	
Lin28	F: 5′-GAA GGG TTC CGG AGC TTG AA-3′
	R: 5′-ACA GTT GTA GCA CCT GTC TCC-3′
Oct4	F: 5′-GTG GAG GAA GCT GAC AAC AA-3′
	R: 5′-GCC GGT TAC AGA ACC ACA CT-3′
Nanog	F: 5′-CTG CAG AGA AGA GTG TCG CA-3′
	R: 5′-ACC AGG TCT TCA CCT GTT TGT-3′
Sox2	F: 5′-GAC AGT TAC GCG CAC ATG AA-3′
	R: 5′-TAG GTC TGC GAG CTG GTC AT-3′
Msi1	F: 5′-GGA GTT ATA CAG GCC TCG CC-3′
	R: 5′-TGA GAG CCT GTC CCT CGA A-3′
*Hepatic growth factor and hepatic growth factor receptor*	
HGF	F: 5′-AGG ACT TCC ATT CAC TTG CAA GGC T-3′
	R: 5′-ACT GTT CC TTG TAG CTG CGT CC-3′
c-MET	F: 5′-GCC TGC AAT CTA CAA GGT TTT CCC A-3′
	R: 5′-AGT CAA GGT GCA GCT CTC ATT TCC-3′

### In vivo Study

Animal study was approved by the Committee on the Use of Live Animals for Teaching and Research of the University of Hong Kong (CULTR no. 2895-12). NOD/SCID mice were maintained in laminar flow cabinets under pathogen-free conditions. CTL and SorR cells, derived from PLC/PRF/5 cells, were harvested from mid-log phase cultures and resuspended in a 50% Matrigel (BD Biosciences) in culture medium. Cells (1×10^7^) were injected under the capsule of the left liver lobe. Mice were sacrificed at week 6. Under anesthesia, D-luciferin (Life Technologies) was injected i.p. and PLC/PRF/5 cells expressing luciferase produced a bioluminance signal, which was detected by the IVIS imaging system 100 (Xenogen, Alameda, CA). An elliptical region of interest (ROI) was placed over the tumors, and the total signal in the ROI (photons per second) was quantified using the Living Image software (Xenogen). Liver and lung were dissected, fixed in 10% formalin, and paraffin-embedded for further analysis.

### Immunohistochemistry (IHC) and Hematoxylin and Eosin (H&E) Staining

The paraffin-embedded tissues were sectioned, deparaffinized and rehydrated through a series of xylenes and ethanol. Antigen retrieval was performed by boiling in sodium citrate buffer (10 mmol/L sodium citrate, pH 6.0). Slides were then incubated with anti-Ki-67 (Dako) or anti-CD44 (Dako) overnight at 4°C and signal was detected by the LSAB+ System-HRP kits (Dako) according to the manufacturer’s instruction. Sections were then counterstained with hematoxylin and dehydrated through a series of ethanol and xylenes.

For the staining of CD44 and Ki-67 of the primary tumors, scoring of staining was performed by two independent investigators who were blinded to the study groups. The scoring was based on the percentage and intensity of the positively stained cells under high power (400X) microscopy. The staining of the protein of interest was graded from 0–3 for intensity (0 =  negative staining, 1 = weakly positive staining, 2 =  moderately positive staining and 3 =  strongly positive staining) and percentage (0 =  negative, 1 =  positive staining in <30% of cells, 2 = 30–70% and 3 =  >70%), respectively. The score of each section was the sum of both parameters. Sections were also stained with Mayer’s hematoxylin and eosin, and analyzed for metastasis.

### Statistical Analysis

Data are presented as means ± SD from three independent experiments for the *in vitro* study and from n = 8 for the *in vivo* study. Data were statistically analyzed with one-way ANOVA, and were considered statistically significant at *p*<0.05.

## Results

### Sorafenib-induced Resistance in HCC Cell Lines

**Figure 1:**
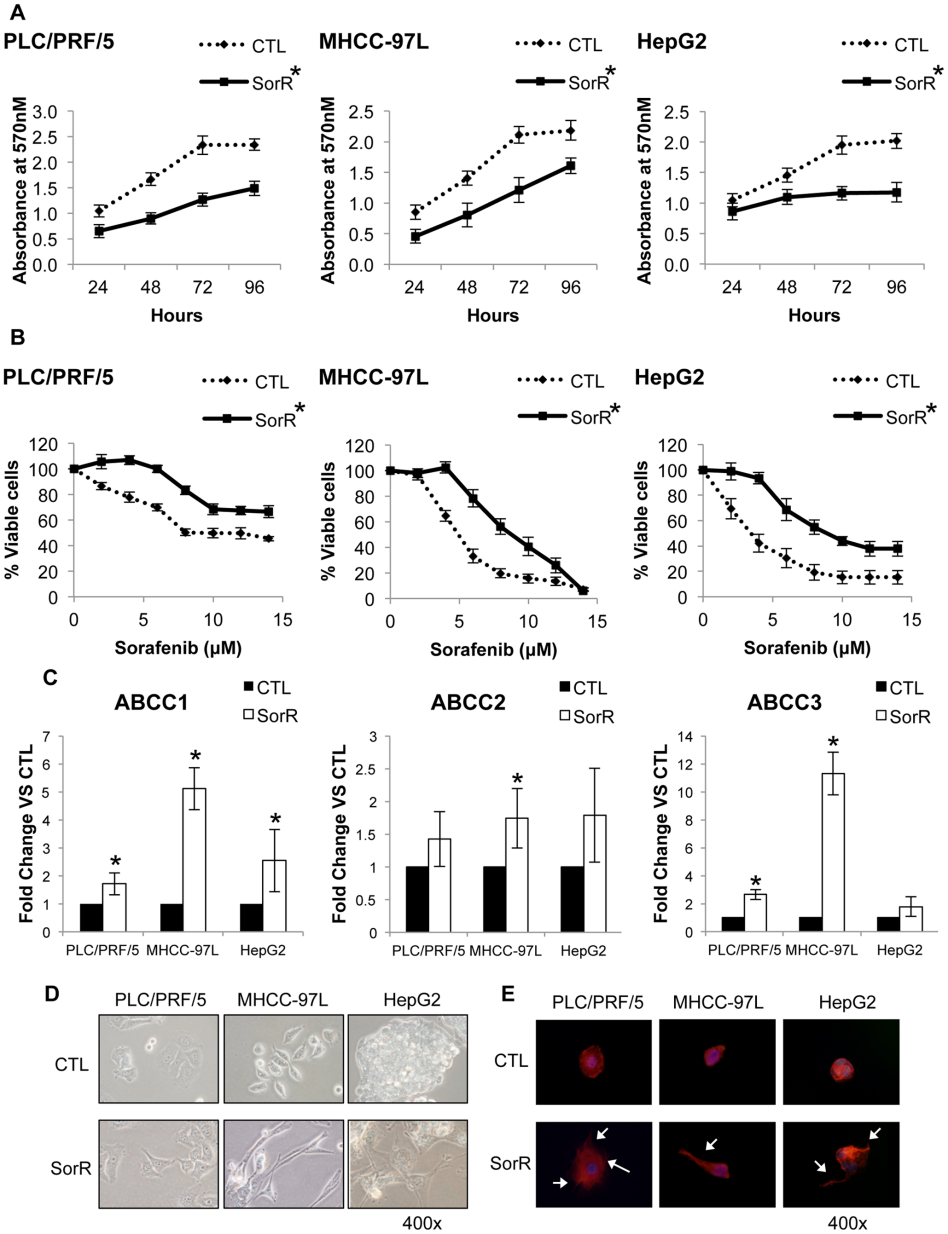
Establishment of SorR cells using HCC cell lines. PLC/PRF/5, MHCC97L and HepG2 cells were cultured at maximal tolerated dose of sorafenib to obtain the CTL and SorR cells derived from each cell line. A) CTL and SorR cells were cultured at 0–14 µM sorafenib and MTT assay was performed 72 hours after treatment. B) Total RNA from CTL and SorR cells were extracted to perform the qPCR analysis of ABCC1, ABCC2 and ABCC3. C) Representing images of CTL and SorR cells under a phase-contrast microscopy (magnification: 400x). D) CTL and SorR cells were stained with phalloidin (red) and counterstained by DAPI (blue). Representing images of CTL and SorR cells under a fluorescence microscopy (magnification: 400x). Cellular protrusions were indicated by arrows. Data are presented as means ± SD from three independent experiments. *p<0.05 vs. CTL cells by one-way ANOVA.

SorR cells were first established by culturing PLC/PRF/5, MHCC-97L and HepG2 cells in medium with increasing concentration of sorafenib. The maximum tolerated dose of sorafenib for PLC/PRF/5, MHCC-97L and HepG2 cells are 6 µM, 7 µM and 4 µM, respectively. MTT assays of different cell lines demonstrated a significant difference of CTL and SorR cells in response towards sorafenib treatment (Fig. 1A) and the IC_50_ value of sorafenib of different cell lines were shown in Table 2. SorR cells demonstrated a higher IC_50_ value of sorafenib than the CTL cells. In addition, SorR cells also showed a higher expression of ABCC1, ABCC2 and ABCC3 than that of the CTL cells (Fig. 1B), which further confirm the presence of drug resistance in SorR cells.

**Table 2:** IC_50_ of CTL and SorR cells towards sorafenib treatment.

	PLC/PRF/5		MHCC-97L		HepG2	
	CTL	SorR	CTL	SorR	CTL	SorR
IC_50_ (µM)	8.04	>14	5.03	8.89	3.72	8.94

Under a phase-contrast microscopy (400×), individual CTL cells demonstrated circular shapes whereas individual SorR cells with flattened and elongated shapes were observed (Fig. 1C). Phalloidin staining of the actin filaments also demonstrated more protrusions of the SorR cells than that of the CTL cells (Fig. 1D).

### Enhanced Cellular Migration and Invasion, and EMT of SorR Cells

**Figure 2:**
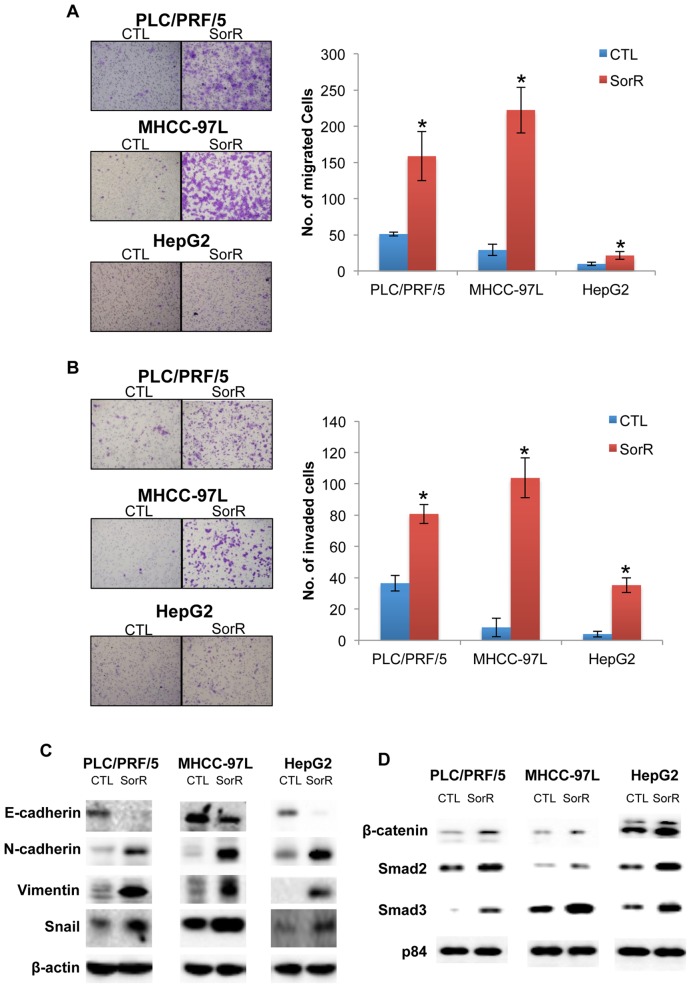
Enhanced cellular migration and invasion with activated EMT process of SorR cells. CTL and SorR cells derived from PLC/PRF/5, MHCC97L and HepG2 cells were plated in top chambers to perform the migration and invasion assay. A) Representing images of the migrated cells under a phase-contrast microscopy (magnification: 100x) were shown in the left panel and the number of migrated cells was counted and presented in the right panel. B) Representing images of the invaded cells under a phase-contrast microscopy (magnification: 100x) were shown in the left panel and the number of invaded cells was counted and presented in the right panel. Data are presented as means ± SD from three independent experiments. *p<0.05 vs. CTL cells by one-way ANOVA. C) Immunoblotting analysis demonstrated the change in total protein expression of E-cadherin, N-cadherin, Vimentin and Snail. The expression level of β-actin was used as loading control. D) Immunoblotting analysis demonstrated the change in protein expression of β-catenin, Smad2 and Smad3 from the nuclear fraction. The expression of nuclear matrix protein p84 was used as loading control.

We further compared the migratory and invasive ability of CTL and SorR cells using the migration and invasion transwell assays. When compared with the CTL cells, SorR cells demonstrated a 3-, 8-, and 2-fold higher in the numbers of migrated PLC/PRF/5, MHCC-97L and HepG2 cells, respectively (Fig. 2A). Similarly, SorR cells showed a 2-, 13-, and 9-fold higher in numbers of invaded PLC/PRF/5, MHCC-97L and HepG2 cells, respectively (Fig. 2B).

In order to explain the enhanced migratory and invasive ability of the SorR cells, immunoblotting on the EMT regulatory proteins, including E-cadherin, N-cadherin, Vimentin, and Snail, and nuclear accumulation of β-catenin, Smad2 and Smad3 were performed (Fig. 2C and 2D). In all cell lines, SorR cells demonstrated a lower expression level of the epithelial marker (E-Cadherin) with higher expression levels of mesenchymal markers (N-Cadherin, Vimentin, and Snail) and a nuclear accumulation of β-catenin, Smad2 and Smad3. These changes in expression levels indicated an activation of the EMT process of SorR cells, which promote the enhanced migration and invasion of SorR cells.

### Enriched CSCs Subpopulations in SorR Cells

**Figure 3:**
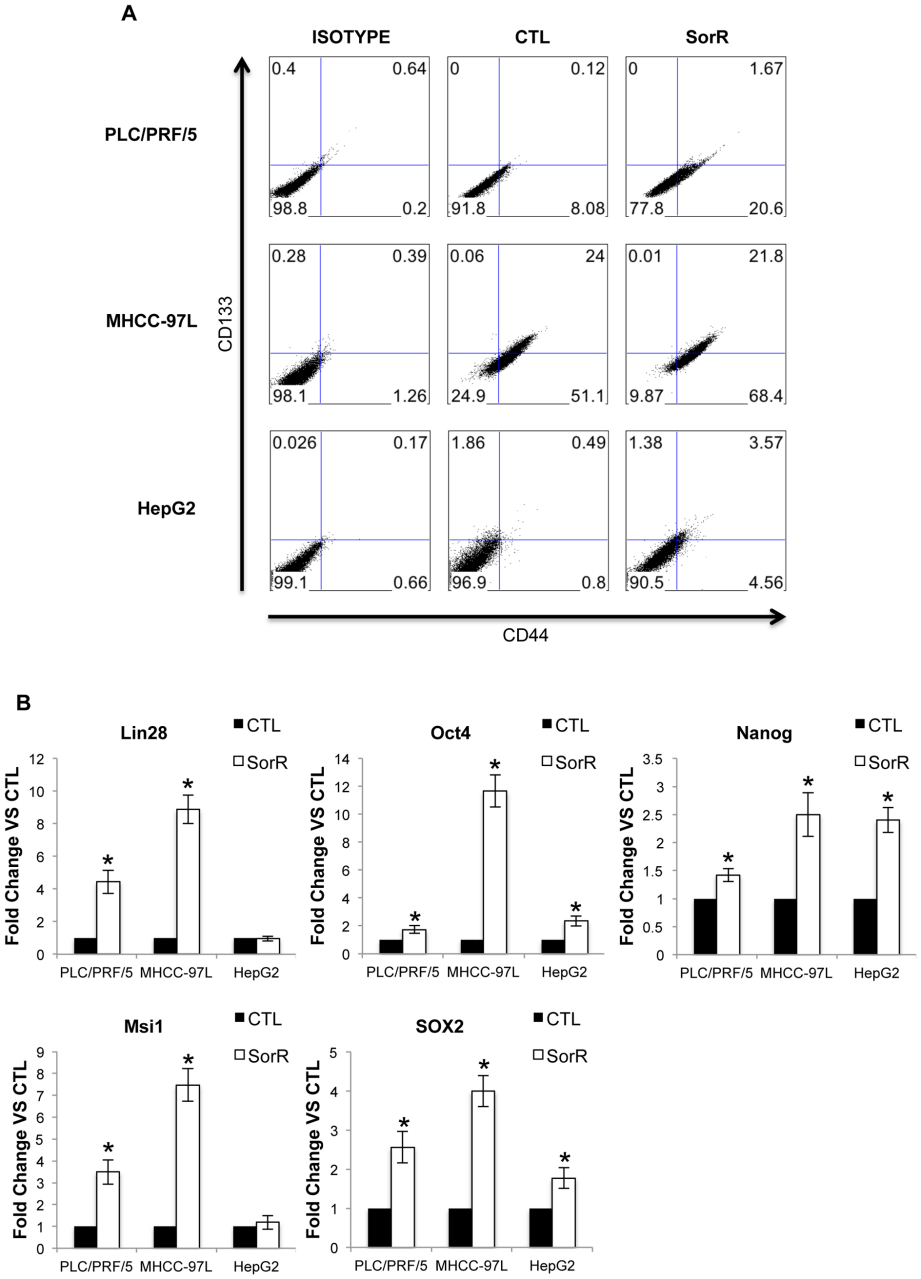
Enriched CSCs subpopulation in SorR cells. A) Harvested CTL and SorR cells derived from PLC/PRF/5, MHCC97L and HepG2 cells were stained with CD44 (FITC) and CD133 (APC) antibodies and 20000 cells were assessed by flow cytometry. The percentage of cells was indicated in each quadrant. B) Total RNA from CTL and SorR cells were extracted to perform the qPCR analysis of Lin28, Oct4, Nanog, Msi1 and SOX2. Data are presented as means ± SD from three independent experiments. *p<0.05 vs. CTL cells by one-way ANOVA.

CD44 and CD133 are commonly used as cell surface markers representing the cancer stem cells (CSCs) subpopulation in HCC [23]. CSCs are resistant to conventional chemotherapy, and therefore we further our study to investigate the enrichment of CSCs in SorR cells using the cell surface marker of CD44 and CD133 (Fig. 3A). SorR cells of all the three cell lines demonstrated a significant enrichment of the CD44^+^ and CD44^+^CD133^+^ subpopulations but not the CD133^+^ subpopulations. In addition, gene expression of the pluripotency factors was studied (Fig. 3B). SorR cells of all the three cell lines demonstrated a significant higher in expression of Lin28, Oct4 Nanog, Msi and SOX2 except no difference was found in the expression of Lin28 and Msi1 in HepG2 cells. These suggested that the enriched CSCs subpopulation in SorR cells.

### Higher Metastatic Potential of SorR Cells

**Figure 4:**
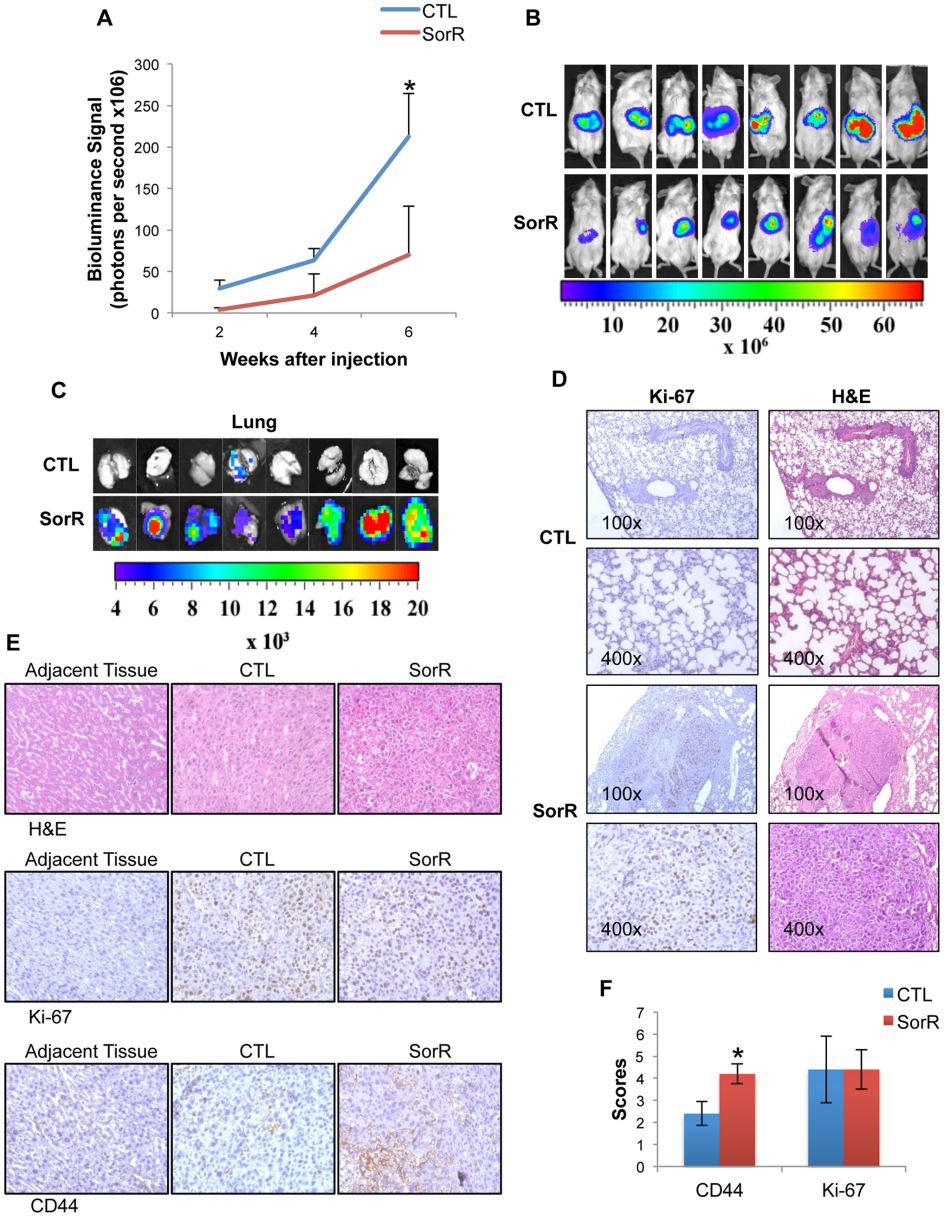
Higher metastatic potential of SorR cells in an orthotopic model. CTL and SorR cells derived from PLC/PRF/5 cells were injected under the capsule of the left liver lobe. A) Under anesthesia, bioluminance signal produced by the injected cells were measured to study the tumor size at week 2, 4 and 6. B) Mice were sacrificed at week 6, bioluminance signal from primary tumor were detected to quantify the tumor size. C) Lung were isolated and bioluminance signal demonstrated the presence of injected cells which represents lung metastasis. D) Representing IHC staining of Ki-67 (left panel) and H&E staining (right panel) the lung sections obtained from CTL and SorR group (magnification: 100x and 400x). E) Representing H&E staining (first row), IHC staining of Ki-67 (second row) and IHC staining of CD44 (third row) of adjacent liver and primary tumor obtained from CTL and SorR group (magnification: 400x). F) The scoring of IHC staining of CD44 and Ki67 based on the percentage and intensity of the positively stained cells under high power (400x) microscopy was performed. Data are presented as means ± SD from 8 mice in each group. *p<0.05 vs. CTL cells by one-way ANOVA.

For the *in vivo* study, orthotopic injection of the CTL and SorR cells to the left liver lobe were done. Unexpectedly, tumor sizes from CTL group were significantly larger than that from the SorR group 6 weeks after injection (Fig. 4A and 4B). We further studied on the metastatic potential of the cells and found that only 1 out of 8 mice demonstrated lung metastasis in CTL group whereas all (8 out of 8) mice demonstrated lung metastasis in SorR group (Fig. 4C). IHC analysis on Ki-67 staining and H&E staining was performed to confirm that the bioluminance signal was originated from the metastatic lung (Fig. 4D). Positive signals of the Ki-67 staining represent the presence of human proliferating cells and only lungs from the SorR group demonstrated a positive signal of the Ki-67 staining. H&E staining also demonstrated a characteristic spongy-like appearance of the lung section obtained from CTL group whereas filling of the alveoli with neoplastic cells were observed in the lung section obtained from SorR group. As the expression of CD133 was undetected by IHC staining (data not shown), only staining of CD44 and Ki-67 of the primary tumors obtained from both CTL and SorR group were shown (Fig. 4E and 4F). No positive signal was obtained for the staining of Ki-67 and CD44 from the adjacent liver tissues of both CTL and SorR groups. When compared with the sections obtained from the primary tumors of the CTL group, sections from the SorR group demonstrated a stronger staining of CD44 and no significant difference in the strength of signal of Ki-67 staining were obtained. This *in vivo* study demonstrated a higher metastatic potential with enriched CD44^+^ subpopulations in the SorR group.

## Discussions

The complex biology of HCC makes it one of the most drug-resistant tumors and intrinsic or acquired drug resistance can easily develop. The development of sorafenib has been a great hope to most advanced HCC patients due to the improved overall survival benefit of sorafenib treatment. Unfortunately, clinical evidence demonstrated the possibility of developing acquired resistance to sorafenib in advanced HCC patients. In this study, long-term exposure to sorafenib has successfully induced sorafenib resistance in different HCC cell lines and both *in vitro* and *in vivo* data demonstrated an increase in metastatic potential of the resistant cells. This suggested that the high metastatic potential of SorR cells is another potential risk of HCC patients who developed sorafenib resistance.

EMT is a critical event in the development of the invasive and metastatic potentials in cancer progression and EMT is initiated by several inducers such as tumor growth factor- β (TGF-β) [24], hepatocyte growth factor (HGF) [25], epidermal growth factor (EGF) and Wnt, through the regulation of Wnt/β-catenin, TGF-β/Smad, and Notch and Hedgehog (Hh) signaling pathways (as reviewed in [26]–[29]). This complex signaling network starts with the cleavage of E-cadherin, which causes adherens junction breakdown and indirect increase in expression of transcription factors, including zinc finger proteins of the snail/slug family and β-catenin. The repression of E-cadherin by snail, or other repressors leads indirectly to increase in expression of N-cadherin, vimentin and other mesenchymal gene products. The cells then acquired a more invasive and metastatic phenotype and numerous studies have demonstrated the correlation of EMT activation with poor prognosis including tumor relapse and metastasis [30]–[32]. The findings of this study is consistent with the results obtained in a very recent study by Malenstein et al stating that long-term exposure of HepG2 cells to sorafenib induces sorafenib resistance with enhanced EMT and increased invasive ability [33]. In this study, we further validate the activation of EMT process through the *in vivo* study that demonstrated the success of lung metastasis.

Besides EMT, presence of CSCs also enhances the invasive and metastatic potentials in cancer progression. CSCs are tumor-initiating cells in the bulk of tumors that possess the ability to self-renewal, divide and differentiate into multiple cell lineages. They are multi-drug resistance and are able to initiate the formation of a new tumor, leading to tumor recurrence and metastasis even after removal of the primary tumor. Systemic chemotherapy is effective in killing differentiated, fast-growing cancer cells. However, it induces chemoresistance and it is well-known that chemotherapy enriches CSCs population which highly increases the risk of recurrence and metastasis. Similarly, in this study, sorafenib resistance was also found to enrich CSCs population. Molecular pathways including TGF-β, Wnt, Notch and Hh that modulate EMT activation were also found in CSCs [34]–[36]. Therefore, activation of the EMT process in SorR cells may also enrich the CSCs subpopulation leading to the enhanced invasive and metastatic potentials. Zhu et al suggested that CD133^+^CD44^+^ cells are subpopulation of cells processing CSCs properties in HCC [23]. CD133^+^CD44^+^ cells demonstrated to process a higher colony forming ability, a greater tumorigenicity in immunosuppressed mice, and are more resistance to doxorubicin treatment when compared with the CD133^+^CD44^−^ counterparts. In this study, the CD44^+^ and CD133^+^CD44^+^ subpopulation of cells were enriched in sorafenib resistant cells. In addition the expression of some pluripotency factors were also enhanced in sorafenib resistant cells and these further suggested the presence of stem-like cells in the resistant cells. These findings further suggested the potential recurrence and metastatic risk in HCC patients with sorafenib resistance.

Some recent *in vitro* studies suggested that treatment with sorafenib retained the levels of expression of ABCC1-3, which were significant induced by treatment with gemcitabine and doxorubicin, and hence sensitizes HCC cells towards doxorubicin and gemcitabine treatments [37]. In another study, Nagai et al suggested that treatment with sorafenib and U0125 (a MEK inhibitor) of Huh7 and HepG2 markedly inhibited the HGF-induced EMT by inhibiting EMT-like morphologic changes, snail expression and E- to N-cadherin switching and thus completely canceled the HGF-mediated cellular migration in HCC cells [38]. However, in this study SorR cells demonstrated a higher expression of ABCC1-3 and an activation of the EMT process. In addition, the RNA expression of HGF but not c-Met in SorR cells is significantly higher than the CTL cells (Fig. S1), which may further facilitate the EMT process. These suggested that inhibition of the EMT process by sorafenib is effective in sorafenib sensitive cells but sorafenib resistant cells retain the power of transition and thus enhance the ability to migrate and invade.

In view of the ease of developing drug resistance towards mono-therapy, an effective cancer therapy may require combination chemotherapy. The combination chemotherapy can help to reduce the chance of developing chemoresistance towards single drug, target on different mechanisms during cancer development to raise the chance of eliminating the cancer, and allow using drugs at lower doses to reduce the chance of having toxic effects of a single drug. Tai et al also demonstrated that STAT-3 is activated in SorR cells and suggested that treatment with dovitinib, another multikinase inhibitor, which effectively decreases phosphorylated STAT3 by increasing SHP-1 activity, can overcome the sorafenib resistance in HCC cells [39]. Another potential biomarker of the sorafenib sensitivity is the activation of the PI3K/Akt pathway and addition of an Akt inhibitor can restore the sorafenib sensitivity in resistant cells. Therefore, by screening the expression of potential markers, which determine the sorafenib sensitivity, one can predict the tumor response towards sorafenib treatment and the addition of a specific inhibitor may help to sensitize the tumors response towards sorafenib. In addition, as both EMT and CSCs share similar molecular pathways, molecular targeted drug targeting on these specific pathways may help to completely eradicate the cancer. Therefore, it is worthwhile to continue the study on combination chemotherapy especially with molecular targeted drugs, which helps to reduce the chance of developing drug resistance to a mono-therapy, and enhance the anti-tumor efficacy through different signaling pathways.

To conclude, long-term exposure of HCC cells with sorafenib induced sorafenib resistance. The sorafenib resistant cells demonstrated an activation of the EMT process with enhanced invasive and metastatic potentials. The proportion of CSCs was also enriched, which further suggested the potential risk of having recurrence and developing distant metastasis in HCC. Currently, sorafenib is the most potent drug prescribed to patients with advanced HCC and it is a common practice for clinicians to continue the sorafenib treatment even though the patients become less responsive towards the treatment. However, this study suggested that this might lead to the development of sorafenib resistance, which increased the chance of developing distant metastasis in HCC patients. A combination chemotherapy was also suggested to prevent the development of sorafenib resistance and enhance the therapeutic potential of the therapy in advanced HCC patients.

## Supporting Information

Figure S1 **Gene expression of HGF and c-MET.** Total RNA from CTL and SorR cells derived from PLC/PRF/5, MHCC97L and HepG2 cells were extracted to perform the qPCR analysis of HGF and c-Met. Data are presented as means ± SD from three independent experiments. *p<0.05 vs. CTL cells by one-way ANOVA.
(TIF)
