# Supplementary figures and images for: The Enhanced Metastatic Potential of Hepatocellular Carcinoma (HCC) Cells with Sorafenib Resistance
Source: PLoS One. 2013 Nov 11;8(11):e78675. doi: 10.1371/journal.pone.0078675 (PMC3823841; doi:10.1371/journal.pone.0078675)

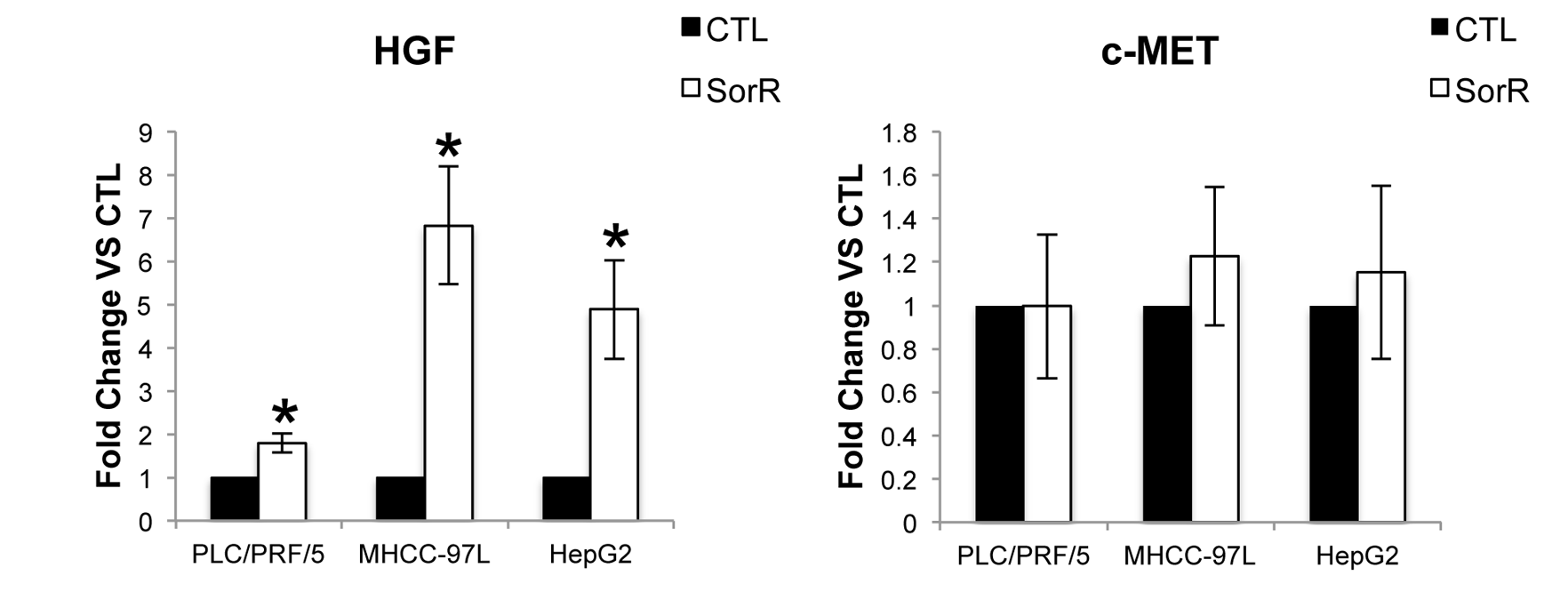

Supplement: Figure S1 — Gene expression of HGF and c-MET. Total RNA from CTL and SorR cells derived from PLC/PRF/5, MHCC97L and HepG2 cells were extracted to perform the qPCR analysis of HGF and c-Met. Data are presented as means ± SD from three independent experiments. *p<0.05 vs. CTL cells by one-way ANOVA. (TIF) [file pone.0078675.s001.tif]
